# Persistent and widening geographic disparities in liver cancer mortality in China, 1987–2021: a 35-year analysis from a national perspective

**DOI:** 10.1016/j.jncc.2025.12.006

**Published:** 2026-01-27

**Authors:** Qianru Li, Siyi He, Mengdi Cao, Yi Teng, Nuopei Tan, Jiachen Wang, Yujie Wu, Tianyi Li, Yuanjie Zheng, Tingting Zuo, Changfa Xia, Wanqing Chen

**Affiliations:** National Cancer Center/ National Clinical Research Center for Cancer/Cancer Hospital, Chinese Academy of Medical Sciences and Peking Union Medical College, Beijing, China

**Keywords:** Liver cancer, Mortality, China, Cancer prevention, Disparity

## Abstract

**Objective:**

Although liver cancer mortality in China has declined overall, long-term trends in geographic and demographic disparities remain poorly understood. This study aimed to evaluate temporal trends and evolving patterns in liver cancer mortality, focusing on subnational disparities and shifts in disease burden.

**Methods:**

Mortality data were obtained from two sources: the World Health Organization (WHO) Mortality Database (1987–2000), which provided nationally representative vital registration data stratified by urban–rural residence; and the Chinese Disease Surveillance Points (DSP) system (2004–2021), a population-based sampling network that gradually expanded to 605 surveillance sites across all 31 provinces by 2013, with continuous stratification by urban–rural and eastern–central–western regions. To assess subnational disparities, we calculated the overall and subgroup-specific temporal trends and compared regional age-standardized mortality rates (ASMRs) with the national level as reference. Age-period-cohort (APC) model was applied to decompose age, period, and birth cohort effects. Poisson models were applied to estimate the period-specific rate ratios of mortality aligning with key phases.

**Results:**

Liver cancer mortality increased modestly until around 2004 and then declined overall through 2021 (average annual percentage change –1.09% [95% CI: –1.25%, –0.93%]), with more pronounced reductions in urban and eastern areas, and slower declines in rural and western areas. Eastern and central regions contributed over 80% of the overall national decline. Males consistently had higher mortality than females, with widening sex disparities observed in rural and western areas. Younger age groups experienced greater declines, while older adults showed slower reductions or even increasing trends. APC analysis showed increased mortality risk with age, while period and cohort effects showed marked improvements, particularly among younger individuals, females, and those in eastern urban areas. In contrast, declines were limited in older age groups and western rural populations. Regional disparities in mortality widened over time, with growing rural–urban and western–eastern gaps observed in recent years.

**Conclusions:**

Liver cancer mortality in China remains geographically and demographically unequal, with burden shifting towards western rural regions, and persistent disparities by sex and age. These findings highlight the urgent need for region-specific and population-targeted prevention strategies, particularly for older adults and underdeveloped regions.

## Graphical abstract

**Figure:**
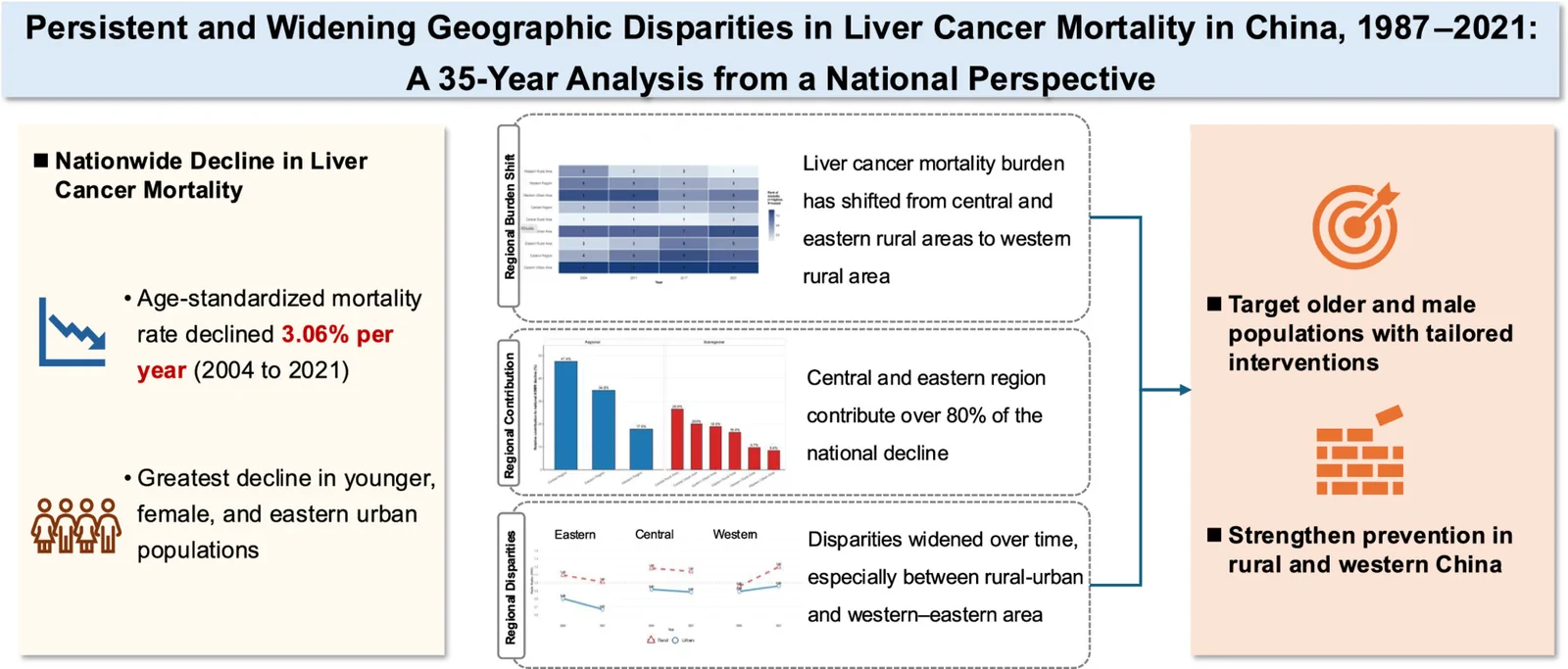

## 1 Introduction

Liver cancer remains a major public health challenge, ranking as the sixth leading cause of cancer-related deaths globally and fourth in China, with an estimated 367,700 new cases and 316,500 deaths in 2022.^1^^,^^2^ Due to its rapid progression and asymptomatic nature in early stages, liver cancer is often diagnosed at advanced stages, resulting in poor prognosis and limited treatment options.^3^ While chronic hepatitis B (HBV) and hepatitis C (HCV) infections, aflatoxin exposure, and alcohol consumption are established risk factors, the vast geographic and socioeconomic heterogeneity across China has contributed to substantial disparities in liver cancer mortality.^4^^,^^5^

Over the past two decades, China has achieved notable progress in reducing liver cancer mortality, largely attributable to the major public health interventions, especially in HBV prevention. The burden of chronic HBV infection was historically high, with a national hepatitis B surface antigen (HBsAg) prevalence of 10.1 % among people under 30 years in 1992, affecting over 20 million individuals with chronic HBV and causing nearly 300,000 deaths annually.^6^ In response, the neonatal hepatitis B vaccine was integrated into Expanded Program of Immunization (EPI) in 2002, and administration fees were then covered by the government from 2005. Moreover, a national catch-up campaign targeting unvaccinated children under 15 was implemented between 2009 and 2011.^7^^,^^8^ These efforts have been demonstrated substantially reducing HBV prevalence and liver-related deaths.^9^^,^^10^

Despite these achievements, regional disparities remain pronounced, with mortality rates remaining higher in rural areas compared to urban areas.^5^ And certain provinces, such as Yunnan, have even experienced increasing mortality trends in recent years.^11^ The subnational disparities might be related to multiple factors, including uneven access to early detection and treatment, variability in health service capacity, environmental exposure and individually lifestyle risk factors.^6^^,^^12^, ^13^, ^14^, ^15^ Although prior studies have described geographic disparities in liver cancer mortality in China, they have largely focused on short-term trends or single time points, with limited attention to the long-term temporal evolution of regional inequalities in relation to policy interventions.^4^^,^^16^ Existing studies on liver cancer mortality trends have primarily focused on broad national or urban-rural aggregates,^17^, ^18^, ^19^, ^20^ without a comprehensive evaluation of regional, sex-, and age-specific disparities across eastern, central, and western China. Moreover, few studies have examined long-term trends simultaneously using population-based data or evaluated the impact of hepatitis B vaccination policies.

To address these knowledge gaps, this study aims to provide a comprehensive, long-term analysis of liver cancer mortality trends in China over three decades, with a particular focus on regional, urban-rural and sex-specific disparities. We utilized data from the World Health Organization (WHO) Mortality Database and China’s Disease Surveillance Points (DSP) system, which together provide a nationally representative and regional stratification information. We also evaluate the relative contributions of age, period, and cohort effects on mortality trends and explored how these effects varied across different population or geographic subgroups. In addition, by examining trends before and after key public health milestones, we explore the potential influence of major HBV control policies. Our study addresses this gap by examining liver cancer mortality patterns from 1987 to 2021 across demographic and geographic subgroups, using age-period-cohort (APC) and Poisson modeling to quantify both structural disparities and policy-associated changes, and to highlight emerging burdens in underserved regions in China.

## 2 Materials and methods

### 2.1 Data sources

Liver cancer mortality data were obtained from two sources: the WHO Mortality Database and China’s DSP system. WHO Mortality Database provides standardized, International Classification of Diseases (ICD)-coded mortality statistics from national vital registration systems, validated through WHO’s Data Quality Assurance Framework.^21^ For China, it contains data from 1987 to 2000, including deaths and population by 5–year age group (0, 1, 5 to 80 by 5–year intervals, and 85+ years), sex (male/female), and area (urban/rural). The DSP system was initially established to monitor national birth and death cases, comprising 145 urban and rural sites and covering approximately 1 % of the national population.^22^ It was subsequently expanded in 2004 and 2013, ultimately including 605 surveillance points across all 31 provinces, covering about 24.3 % of the national population.^23^ The DSP system provided liver cancer mortality data for the years 2004–2021, categorized by 5-year age groups (0, 1–4, 5 to 80 by 5-year intervals, and 85+ years), sex (male/female), and area (urban/rural, and eastern/central/western regions).

Urban and rural classifications in the DSP system were based on administrative divisions, where counties (including county-level cities) were defined as rural areas, and municipal districts were defined as urban areas. Regional classification followed the official standards of the National Bureau of Statistics: the eastern region includes Beijing, Tianjin, Hebei, Liaoning, Shanghai, Jiangsu, Zhejiang, Fujian, Shandong, Guangdong, and Hainan; the central region includes Shanxi, Jilin, Heilongjiang, Anhui, Jiangxi, Henan, Hubei, and Hunan; and the western region includes Inner Mongolia, Guangxi, Chongqing, Sichuan, Guizhou, Yunnan, Xizang, Shaanxi, Gansu, Qinghai, Ningxia, and Xinjiang.^24^ Liver cancer was classified according to the ICD, 9th Revision (ICD-9) codes 155 and 230.8 in the WHO Mortality Database and the 10th Revision (ICD-10) code C22 in the DSP system, respectively.

### 2.2 Statistical analysis

#### 2.2.1 Epidemiological trends and annual percentage change across regions

The annual crude mortality rate of liver cancer was calculated using cases divided by the corresponding population for overall and different regions by sex, 5-year age group. The age standardized mortality rate (ASMR) was calculated using the direct standardization methods with the national population in 2010 as a reference.^25^ The standardized rate ratio (SRR) between males and females was calculated by using ASMR of males divided by ASMR of females. To assess the long-term temporal trends in liver cancer mortality, we employed segmented regression analysis using the segmented package in R to estimate the average annual percent change (AAPC) from 1987 to 2021, and for period from 1987 to 2000 and period for 2004 to 2021, separately.^26^ Breakpoints were automatically selected by the model, with the maximum number limited to three to avoid overfitting and ensure interpretable trends. Trends were summarized using AAPC estimates to determine changes over time. An AAPC > 0 indicates an increasing trend, whereas an AAPC < 0 indicates a decreasing trend. To assess subnational disparities, ASMRs of different regions were compared against the national level to calculate geographic rate ratios. Furthermore, to quantify regional contributions to national mortality decline, contribution to the national ASMR reduction of each region was calculated by weighting changes between 2004 and 2021.

#### 2.2.2 Age-Period-Cohort model analysis

The Age-Period-Cohort model between 2004 and 2021 was employed to assess the effect of age, period and cohort across different regions.^27^ Analysis was restricted to individuals aged ≥ 25 as few deaths records were observed in DSP under age 25. The model fits a log-linear Poisson regression to a Lexis diagram, with the general form:

$$\text{log}\left(\lambda_{apc}\right)=\mu +\alpha_{a}+\beta_{p}+\gamma_{c}$$

Where $\lambda_{apc}$ represents the mortality rate, μ the mean effect of age, period, and cohort, and $\alpha_{a}$, $\beta_{p}$, $\gamma_{c}$ the age, period, and cohort effects, respectively. Due to the exact linear dependency among these three variables (cohort = period − age), the model faces identifiability problem which makes it impossible to estimate all effects simultaneously.^28^ To address this, we decomposed the mortality into overall linear drifts. Net drift was interpreted as the overall log-linear trend across periods and cohorts, representing the estimated annual percent change in mortality over time. Local drift referred to age-specific annual percent changes, reflecting potential cohort-related changes within each age group.

For cohort estimation, individuals were grouped into twelve 5-year age categories. Birth cohorts were calculated by subtracting the median age of each group from the study year. Periods were categorized into four intervals: 2004–2006, 2007–2011, 2012–2016, and 2017–2021. Age effects were represented as the age-specific rates in the reference cohort after controlling for period and cohort effects, with the period function constrained to be 0 on average with 0 slope.^29^ Longitudinal age curves were generated to depict age-specific mortality patterns. Period and cohort effects were presented as relative risks (RRs), compared with reference categories (2014 and 1958 as the period and birth cohort, respectively). Natural spline functions with three to seven knots were used to parameterize age, period, and cohort effects to evaluate the robustness of the models. The significance of estimable parameters and functions was tested using Wald χ² statistics.

#### 2.2.3 Multivariable regression analysis

To evaluate the long-term effects of national control strategies on liver cancer mortality, the study period was separated into four phases based on major policy milestones: including Period 1, 1987–1991 (pre-vaccination era, before the HBV vaccine was introduced into the national immunization schedule); Period 2, 1992–2002 (early promotion phase, when infant HBV vaccination was recommended but not yet universally provided free of charge); Period 3, 2003–2011 (expansion phase, during which HBV vaccination was incorporated into the national EPI, catch-up campaigns were initiated, and rural cancer screening programs were piloted); and Period 4, 2012–2021 (integrated control phase, characterized by improved vaccination coverage and broader implementation of national cancer screening and control strategies). To examine potential age-related differences in intervention effects, the population was categorized into four age groups: Children and adolescents (< 30 years), young adults (30–49 years), middle-aged adults (50–69 years), and older adults (≥ 70 years).

A multivariable Poisson regression model was applied to estimate the RRs of liver cancer mortality across periods, adjusting for region and sex. The results are presented with the RR with confidence interval (CI), and the percentage of change was calculated as (RR–1) × 100 %. Comparisons were made between Periods 1 and 2 and Periods 2 and 3, respectively. Model fit was assessed using residual deviance and degrees of freedom. Overdispersion was evaluated by computing the Pearson chi-squared dispersion statistic. More details about the model methods could be found in other publications.^6^

Additional sensitivity analyses including segmented regression and interrupted time-series (ITS) models were applied to identify data-driven breakpoints and to test for potential discontinuities around the 2004 data source transition. Detailed model specifications and results are provided in the Supplementary materials. All analyses were performed using R (version 4.3.2), and *P*-values < 0.05 were considered statistically significant.

## 3 Results

### 3.1 National trends and regional variations

**Fig. 1:**
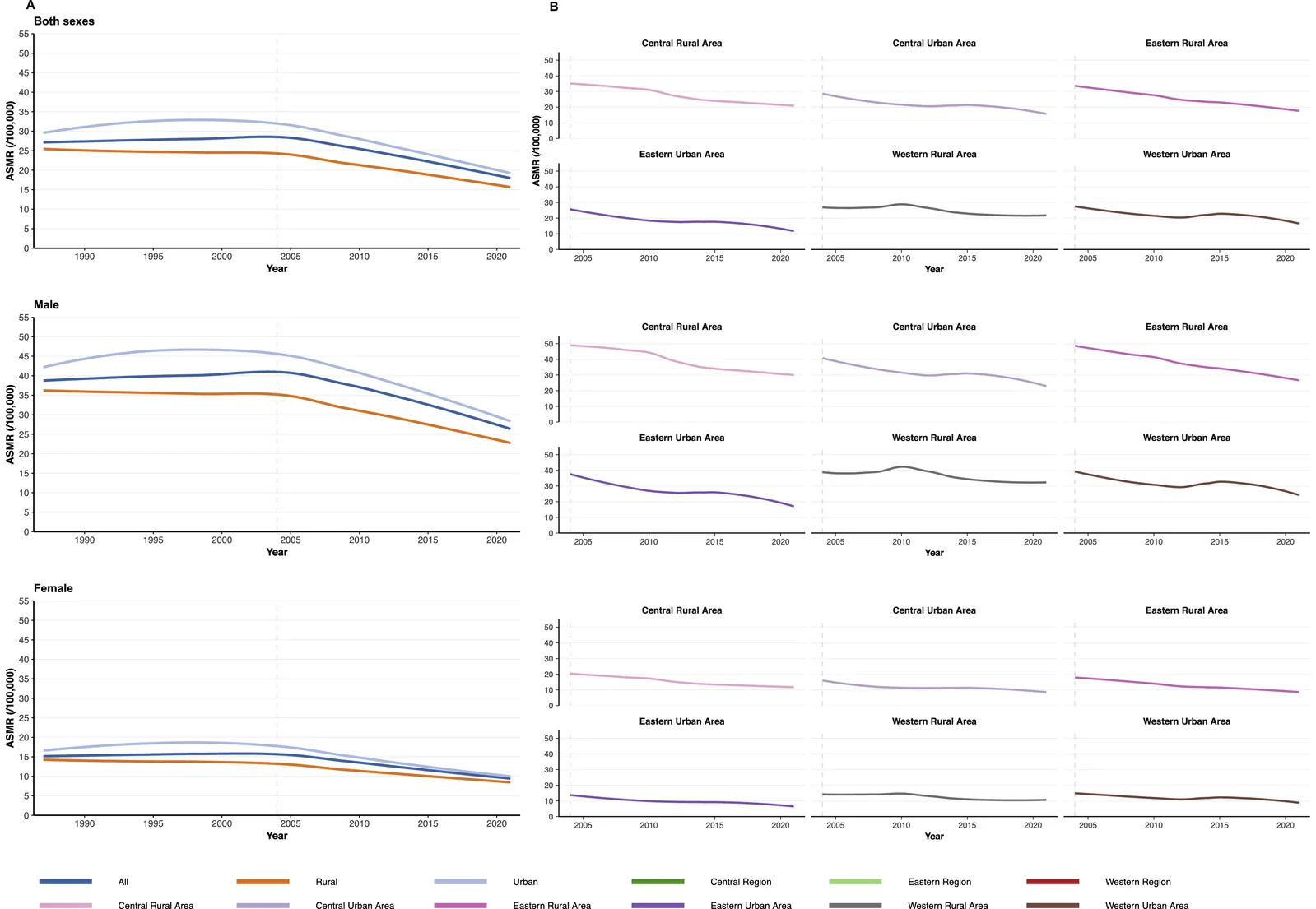
Age-standardized mortality rates of liver cancer in China, by sex and geographic characteristics. (A) National and urban–rural trends combining World Health Organization and DSP data (1987–2021). (B) Subregional urban–rural trends within each region based on the DSP system (2004–2021). ASMR, age-standardized mortality rate; DSP, Chinese Disease Surveillance Points.

From 1987 to 2021, the ASMR of liver cancer mortality in China showed an overall decreasing trend (AAPC, –1.09% [95% CI: –1.25%, –0.93%]) (Fig. 1A and Table 1). The national ASMR peaked around the 2004 and declined steadily thereafter. For different period analysis, the ASMR of liver cancer showed an overall increasing trend from 1987 to 2000, but only statistically significant in males (AAPC, 0.52% [95% CI: 0.03%, 1.02%]) (Supplementary Table 1). From 2004 to 2021, the ASMR showed rapidly decreasing trends overall (AAPC, –3.06% [95% CI: –3.64%, –2.47%]) and across most subgroups (Table 1).

**Table 1:** The annual average percent changes of age-standardized liver cancer mortality rates across regions in China, overall and by sex.

	Both (95 % CI)	*P*-value	Female (95 % CI)	*P*-value	Male (95 % CI)	*P*-value
1987–2021^a^						
Nationwide	−1.09 (−1.25, −0.93)	< 0.001	−1.35 (−1.52, −1.17)	< 0.001	−1.00 (−1.17, −0.84)	< 0.001
Urban	−1.32 (−1.55, −1.09)	< 0.001	−1.49 (−1.71, −1.26)	< 0.001	−1.27 (−1.50, −1.03)	< 0.001
Rural	−1.22 (−1.42, −1.03)	< 0.001	−1.50 (−1.71, −1.28)	< 0.001	−1.11 (−1.32, −0.91)	< 0.001
2004–2021^b^						
Nationwide	−3.06 (−3.64, −2.47)	< 0.001	−3.38 (−4.06, −2.71)	< 0.001	−2.76 (−3.28, −2.25)	< 0.001
Urban	−3.11 (−3.99, −2.22)	< 0.001	−3.21 (−4.00, −2.41)	< 0.001	−3.07 (−3.94, −2.18)	< 0.001
Rural	−2.82 (−3.41, −2.22)	< 0.001	−3.51 (−4.33, −2.67)	< 0.001	−2.62 (−3.22, −2.02)	< 0.001
Eastern egion	−3.51 (−4.03, −2.98)	< 0.001	−3.93 (−4.68, −3.17)	< 0.001	−3.39 (−3.84, −2.95)	< 0.001
Central region	−3.00 (−3.45, −2.56)	< 0.001	−3.47 (−4.06, −2.87)	< 0.001	−2.93 (−3.38, −2.48)	< 0.001
Western region	−1.52 (−2.53, −0.49)	0.004	−2.00 (−2.98, −1.01)	< 0.001	−1.29 (−2.32, −0.25)	0.015
Eastern urban area	−3.92 (−4.58, −3.25)	< 0.001	−3.92 (−4.66, −3.16)	< 0.001	−3.93 (−4.57, −3.29)	< 0.001
Central urban area	−3.03 (−3.95, −2.11)	< 0.001	−3.25 (−4.13, −2.36)	< 0.001	−2.91 (−3.85, −1.96)	< 0.001
Western urban area	−2.47 (−3.93, −0.98)	0.001	−2.56 (−4.18, −0.91)	0.002	−1.82 (−3.01, −0.62)	0.003
Eastern rural area	−3.66 (−4.18, −3.14)	< 0.001	−4.09 (−4.79, −3.38)	< 0.001	−3.30 (−3.71, −2.89)	< 0.001
Central rural area	−3.26 (−3.85, −2.67)	< 0.001	−3.36 (−3.99, −2.72)	< 0.001	−3.06 (−3.73, −2.39)	< 0.001
Western rural area	−1.27 (−2.45, −0.07)	0.038	−1.99 (−3.12, −0.84)	< 0.001	−0.96 (−2.16, 0.26)	0.121

a 1987–2021: Estimates based on combined data from the WHO Mortality Database (1987–2000) and the China Disease Surveillance Points (DSP) system (2004–2021); applied to national, urban, and rural stratifications.

b 2004–2021: Estimates derived solely from the DSP system, which provides more granular geographic detail; used for regional and urban–rural subgroup analyses within eastern, central, and western China. Abbreviation: CI, confidence interval.

**Fig. 2:**
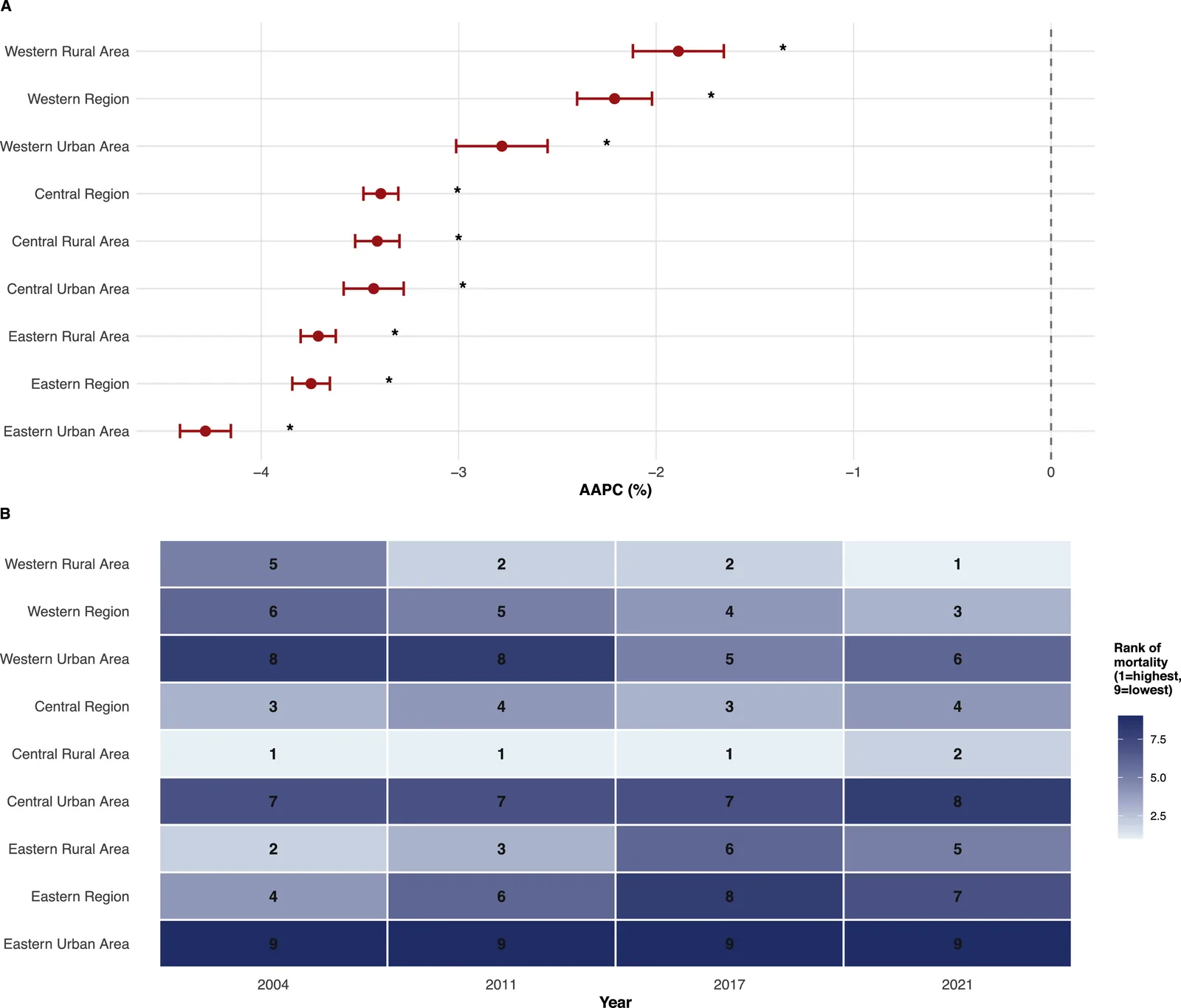
Regional disparities in liver cancer mortality trends and rankings in China, 2004–2021. (A) AAPC in age-standardized mortality rates of liver cancer across nine regions, with 95 % CI. (B) Ranking of liver cancer mortality rates across regions from 2004 to 2021, where a higher rank indicates higher mortality. * denotes statistically significant trends (*P* < 0.05). AAPC, annual average percent change; CI, confidence intervals.

However, substantial geographic disparities were observed in liver cancer mortality trends. Over the entire study period, urban regions experienced a slightly greater decline in liver cancer mortality compared to rural regions (AAPC: –1.32% vs. –1.22%) (Table 1, Fig. 1A). From 1987 to 2000, the ASMR of liver cancer in urban regions showed decreasing trends for both sexes combined (AAPC, –0.44% [95% CI: –0.92%, 0.04%]) and females (AAPC, –0.58% [95% CI: –0.92%, –0.23%]) (Supplementary Table 1). Whereas in rural China, the ASMR still exhibited increasing trends for both sexes (AAPC: 1.21% for females and 1.04 % for males) and combined (AAPC, 1.00%). From 2004 to 2021, eastern region experienced the most pronounced decline in ASMR, whereas the decline in western rural regions was limited (Table 1 and Fig. 1B). From 2004 to 2021, the highest liver cancer mortality shifted from central and eastern rural areas to western rural regions, while eastern urban areas consistently reported the lowest ASMRs (Fig. 2A and B).

**Fig. 3:**
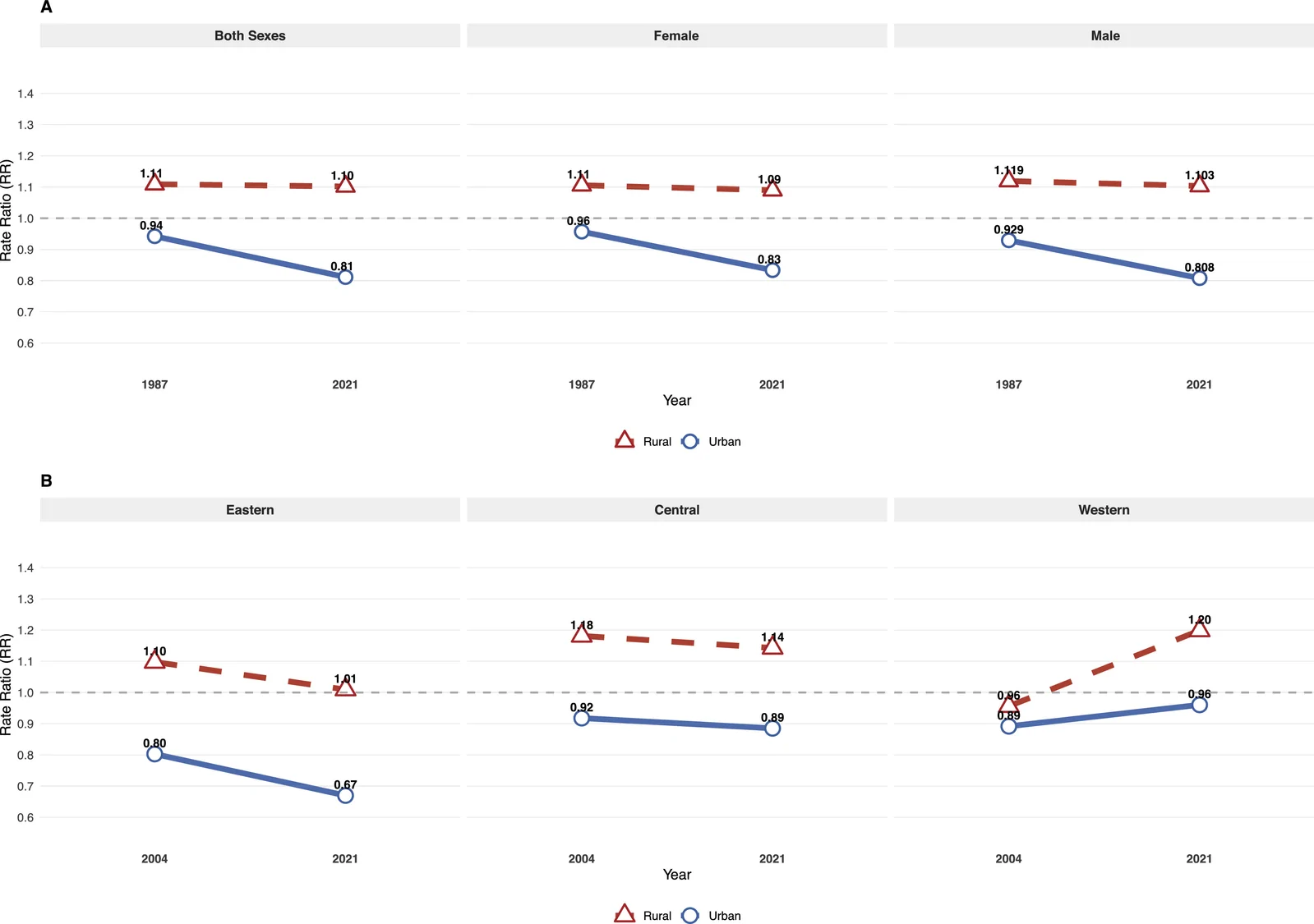
Urban–rural disparities in liver cancer mortality in China, 1987–2021. (A) RRs comparing rural and urban areas by sex (1987 vs 2021). (B) RRs by region (eastern, central, and western) comparing 2004 vs 2021. RRs are relative to the national average (reference = 1.0). RRs, rate ratios.

Moreover, the RR for urban and rural areas from 1987 to 2021 showed a general enlarged gap in recent years, the same findings were found then comparing eastern, central and western region (Fig. 3). The relative contributions of each region to the national ASMR decline from 2004 to 2021 were further quantified (Supplementary Fig. 1), showing that central and eastern regions accounted for over 80 % of the overall reduction, with central rural and urban areas being the largest contributors. Regional and urban–rural disparities in liver cancer mortality were statistically significant across all models (*P* < 0.001; Supplementary Table 2).

### 3.2 Sex-specific trends across regions

Throughout the study period, liver cancer mortality remained consistently higher in males than in females. Females had a more dramatic decrease than males (AAPC: –1.35% vs –1.00%) (Table 1). For regional disparities, the AAPC in the eastern urban areas reached –3.92% for females and −3.93% for males, significantly greater than in western rural areas (AAPC: –1.99% for females and –0.96% for males) (Table 1). The urban–rural differences were more pronounced among males (AAPC: –1.27% in urban areas vs. –1.11% in rural areas), whereas the trends were nearly identical among females (AAPC: –1.49% in urban areas vs. –1.50% in rural areas) (Table 1).

The male-to-female standardized mortality rate ratio ranged from 2.54 to 3.10 across different regions in 2021(Supplementary Fig. 2). Overall, the SRR exhibited an increasing trend from 1987 to 2021, with the most pronounced rise observed in rural areas of western and eastern China. From 1987 to 2000, in urban areas, compared with stable trend for males, the ASMR in females presented decreasing trends (AAPC: –0.58 [95% CI: –0.92, –0.23]). Sex differences in liver cancer mortality were also statistically significant across all models (*P* < 0.001; Supplementary Table 2).

### 3.3 Age-specific trends across regions

Liver cancer mortality rates across all age groups in China showed a general declining trend. The middle-aged groups (35–45 years) showed the most significant decreases in liver cancer mortality rates (Supplementary Fig. 3). Females aged 40–44 years exhibited the most marked decrease nationwide, with an AAPC of –6.11% (95% CI: –8.85%, –3.29%). Analysis of age-specific mortality rates showed that the decrease trends were more obvious in the younger age groups than in the older. The eastern region demonstrated the greatest decline in liver cancer mortality, followed by the central region, while the western region showed the least decline or near-stable trends in some age groups. Particularly, eastern urban areas had the largest reductions, whereas western rural areas exhibited minimal changes.

In the < 30 age group, the trends of liver cancer mortality were different from those of the three other age groups. The mortality rate reached an obvious decrease in the young age group, especially in the urban region (Supplementary Fig. 4). It is obvious that the liver cancer mortality rate peaked later in the oldest (≥ 70) than the youngest throughout the whole period, which peaked in Period 3 and declined thereafter.

### 3.4 Age-period-cohort effects in different regions

**Fig. 4:**
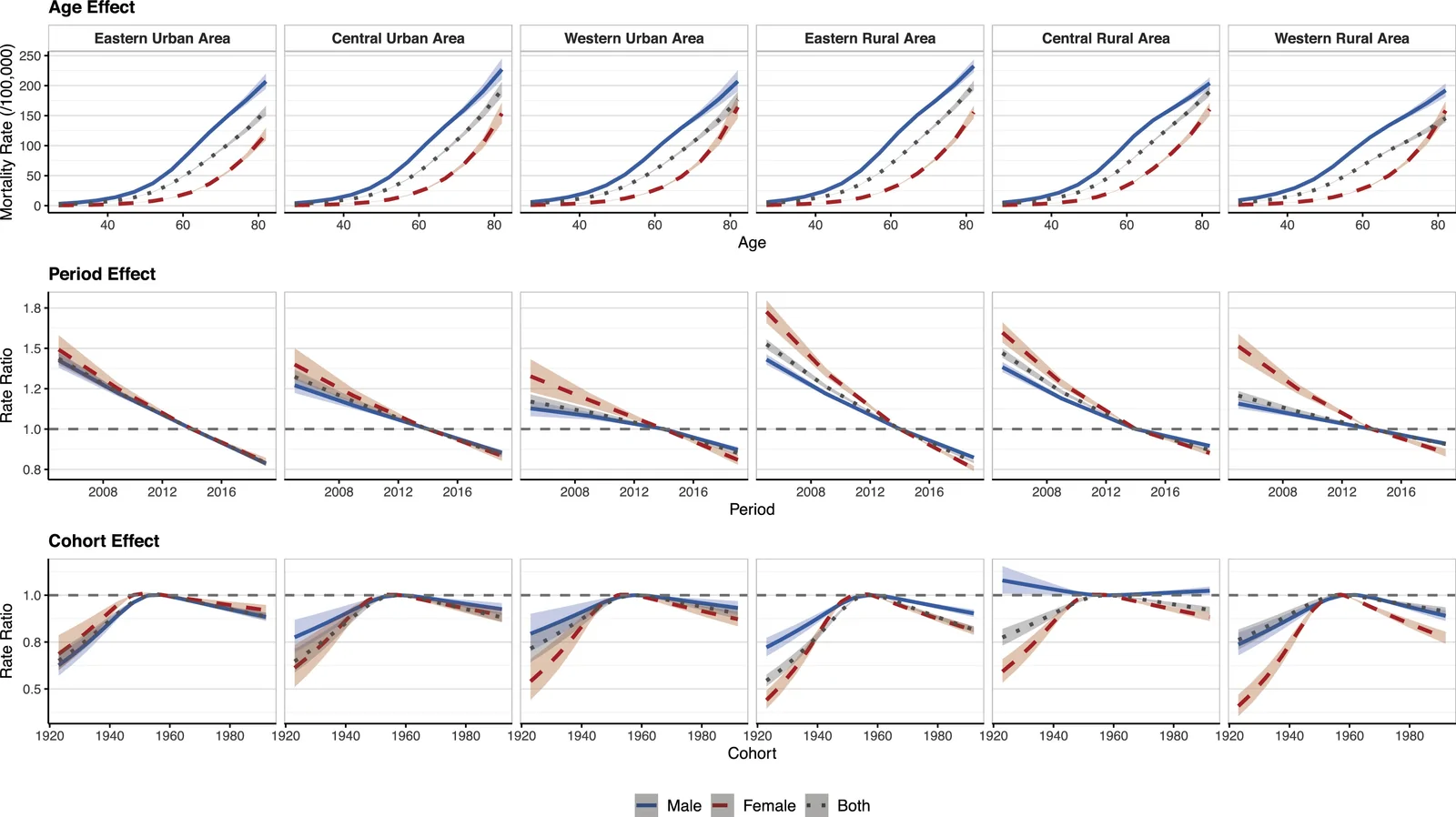
Age, period, and cohort effects on liver cancer mortality from the age–period–cohort model based on Disease Surveillance Points (DSP) data (2004–2021), stratified by region, area, and sex. Shaded bands indicate 95% confidence intervals and the dashed line indicates rate ratio = 1 (reference).

The age effect on liver cancer mortality continuously increases with age among the whole population consistent across regions and sexes (Fig. 4). Mortality rates rose rapidly from approximately aged 40 and peaked in the oldest age groups, with consistently higher age-specific risks observed among males compared to females. The period effect showed a generally decreasing trend from 2004 to 2021 across all areas, with most substantial reductions observed in eastern urban and rural areas. The decline was present in both sexes but appeared more pronounced in females. The cohort effect revealed substantial generational differences in liver cancer mortality. Individuals born in earlier cohorts exhibited higher risks, while those in more recent cohorts experienced progressively lower risks. These declining cohort-associated risks were more prominent among younger cohorts, females and urban eastern populations (Supplementary Fig. 5).

### 3.5 Effects of national progress on liver cancer mortality

Compared with Period 1, liver cancer mortality increased 5%, 7%, and decreased 5% during Period 2, 3 and 4 across all age groups, respectively (Table 2). However, substantial age-specific differences were observed. In < 30 age group, liver cancer mortality decreased successive periods compared to Period 1: by 6% (adjust RR [aRR], 0.94 [95% CI: 0.91–0.96]) for Period 2, 38% (aRR=0.62, 95% CI: 0.60–0.64) for Period 3, 56% (aRR, 0.44 [95% CI: 0.43–0.45]) for Period 4, respectively. For the 30–49 age group, using period 2 as the baseline, the mortality decreased by 27% in Period 3 and 49% in Period 4, respectively. Across all age subgroups, a reduction in mortality was observed in Period 4 compared with Period 1. However, the magnitude of decline was greater among younger individuals, with progressively smaller reductions observed in older age groups, particularly those aged ≥ 70 years. Consistent with these findings, age- and cohort-stratified mortality trends (Supplementary Fig. 5) showed a more pronounced and sustained decline in younger and female populations, particularly in eastern and central urban areas.

**Table 2:** The relative risk for liver cancer mortality rates in China by age and period, adjusted for sex and region.

Age, years	Period	aRR (95% CI)	aRR (95 % CI)	aRR (95 % CI)
All	1987–1991^a^	1 (ref)		
	1992–2002^b^	1.05 (1.05, 1.06)	1 (ref)	
	2003–2011^c^	1.07 (1.07, 1.07)	1.02 (1.01, 1.02)	1 (ref)
	2012–2021^d^	0.95 (0.95, 0.95)	0.90 (0.90, 0.90)	0.89 (0.89, 0.89)
< 30	1987–1991	1 (ref)		
	1992–2002	0.94 (0.91, 0.96)	1 (ref)	
	2003–2011	0.62 (0.60, 0.64)	0.66 (0.64, 0.68)	1 (ref)
	2012–2021	0.44 (0.43, 0.45)	0.47 (0.46, 0.48)	0.71 (0.69, 0.73)
30–49	1987–1991	1 (ref)		
	1992–2002	1.03 (1.03, 1.04)	1 (ref)	
	2003–2011	0.76 (0.75, 0.76)	0.73 (0.73, 0.74)	1 (ref)
	2012–2021	0.52 (0.52, 0.53)	0.51 (0.50, 0.51)	0.69 (0.69, 0.70)
50–69	1987–1991	1 (ref)		
	1992–2002	0.95 (0.94, 0.95)	1 (ref)	
	2003–2011	0.90 (0.89, 0.90)	0.95 (0.95, 0.96)	1 (ref)
	2012–2021	0.71 (0.71, 0.72)	0.76 (0.75, 0.76)	0.79 (0.79, 0.80)
≥ 70	1987–1991	1 (ref)		
	1992–2002	1.07 (1.06, 1.08)	1 (ref)	
	2003–2011	1.21 (1.20, 1.22)	1.13 (1.12, 1.14)	1 (ref)
	2012–2021	0.95 (0.94, 0.96)	0.89 (0.88, 0.89)	0.79 (0.78, 0.79)

a 1987–1991: Pre-vaccination period (before HBV vaccine was introduced into the national immunization schedule).

b 1992–2002: Infant HBV vaccination introduced but not yet universally provided free of charge.

c 2003–2011: HBV vaccination included in the national Expanded Program on Immunization (EPI); catch-up campaigns initiated.

d 2012–2021: Post-catch-up period with improved coverage and wider implementation of HBV control measures. Abbreviations: aRR, adjusted relative risk; CI, confidence interval.

## 4 Discussion

This study provides a comprehensive analysis of long-term trends in liver cancer mortality in China over the past three decades with a focus on evolving geographic patterns from both national and subnational perspectives. While an overall decline has been observed nationwide, our findings highlighted substantial and widening geographic disparities masked by the national improvement. The most significant reduction occurred in eastern urban areas, particularly among younger and female populations, whereas western rural regions experienced minimal improvements or even worsening trends in some subgroups. Notably, the burden of liver cancer has shifted from historically high-burden areas in the eastern and central rural to western rural areas in recent years. And a growing divergence for rural versus urban, and for western versus eastern regions also widened during the study period.

The observed mortality decline in liver cancer was consistent with findings from previous studies,^17^, ^18^, ^19^ and likely attributed to both demographic transitions and implementation of a series of public health interventions. The observed accelerated decline in the post-2004 period corresponds closely with the rollout of national HBV-related policies, including newborn vaccination, integration into the national immunization program and catch-up programs. Notably, our age-specific analysis demonstrates that the most pronounced benefits were concentrated among younger individuals, consistent with their direct exposure to early-life vaccination efforts. In contrast, older adults who missed these interventions showed attenuated mortality reductions, highlighting a lagged policy effect across birth cohorts. The national immunization introduced in 2002, have significantly reduced HBV transmission, particularly among younger cohorts. Nationwide serological surveys have shown a dramatic decline in HBsAg positivity in the general population, from 9.72% in 1992 to below 5.86% in 2020, with the most pronounced improvements observed in children aged 1–4 years.^30^ These results reinforce the importance of universal HBV vaccination in altering the long-term burden of liver cancer through both direct protection and herd immunity.^31^

However, these improvements have not been uniformly distributed across regions. Historically, liver cancer high-risk areas predominantly were concentrated in eastern coastal provinces, such as Jiangsu, Zhejiang, Fujian, and Guangdong with high liver cancer mortality reported in the late 20th century.^32^ However, with the implementation of region-specific interventions starting from the 1980s and 1990s, such as pilot HBV vaccination programs, food safety measures, and improved screening, the liver cancer burden in these traditional high-risk areas has declined steadily.^13^^,^^33^ Our results confirm these trends, showing a sharp decrease in RR in eastern urban areas in recent years. Eastern urban areas, benefiting from stronger public health infrastructure, achieved earlier and more complete HBV vaccine coverage, especially among newborns, which contributed to the earlier onset of mortality reduction compared to other regions.^34^ In contrast, rural areas in the eastern region, despite belonging to economically developed provinces, have not demonstrated the same level of improvement. Our results highlight that rural eastern region, despite being located within economically advanced provinces, did not exhibit the same pace of mortality reduction as their urban counterparts. Moreover, the National Cancer Early Diagnosis and Early Treatment Programme in rural areas initiated in 2005 has been limited to selected counties and applied inconsistently across provinces.^35^ Many rural and western regions also face severe limitations in access to timely diagnosis, clinical treatment, and palliative care services.^36^

Our study revealed a persistent and, in some regions, widening disparity in liver cancer mortality between males and females, particularly in rural and western China. Males are significantly more likely to engage in high-risk behaviors such as obesity, smoking and heavy alcohol consumption, which may exacerbate liver cancer risk independently or synergistically with HBV infection.^37^^,^^38^ Moreover, studies have suggested that estrogen exerts a protective effect against the development of hepatocellular carcinoma in premenopausal women under physiological conditions.^39^ These findings may indicate that males in underserved regions face increased vulnerability due to a combination of higher behavioral risk exposure and potentially reduced access to preventive health services.

Age-related differences in liver cancer mortality also varied significantly across regions. The elderly population in western and rural areas experienced a slower decline or even an increase in certain age groups. This may be explained by cumulative exposure to risk factors, higher comorbidity burden, and age-related decline in physiological resilience.^40^ Furthermore, while younger cohorts benefited substantially from early-life HBV vaccination and improved healthcare access, older male populations, especially in underserved rural and western areas, may have missed these public health advances and remain at disproportionately high risk. Targeted strategies aimed at improving long-term management of chronic HBV infection among older adults are urgently needed, particularly in underserved regions.

Collectively, these findings highlight a dynamic epidemiological shift in liver cancer burden. While once concentrated in high-incidence eastern coastal regions, the burden is now increasingly shifting toward older males in rural and western China. This evolving pattern underscores the need for precision prevention strategies. Future prevention strategies should incorporate sex- and region-specific approaches. This includes expanding HBV vaccination coverage, scaling up antiviral therapy access, and strengthening early detection and treatment services, particularly in rural and remote areas. Tailored health promotion campaigns that address behavioral risk factors among males and promote earlier screening among the elderly.

This study utilized two nationally representative mortality data sources, enabling consistent and robust estimates of over 35-year period trends and regional disparities comparisons on liver cancer mortality. Nevertheless, several limitations should be noted. First, liver cancer mortality data were unavailable for the years 2001 to 2003, as the system ceased nearly all operations from this period because of DSPs adjustment,^41^ which may affect the trend analysis results. To address this concern, we conducted a sensitivity analysis and found that the results remained largely consistent (Supplementary Table 3–5). Moreover, differences in diagnostic criteria, cause-of-death certification practices, and population representativeness over time may introduce bias. As the data span two distinct periods, the observed trends may not be directly comparable across periods. The interpretation of the overall long-term trend should be made with caution and in the context of temporal differences. Additionally, the DSP system includes fewer monitoring sites in western regions, potentially leading to underestimation of mortality in those areas. The urban–rural classification, though consistent, is based on administrative divisions and may not fully capture true socioeconomic or healthcare access differences. Despite these limitations, the two data sources remain the most comprehensive datasets available for such an extended time span, offering important insights into the evolving burden of liver cancer in China.

## 5 Conclusions

Liver cancer mortality in China has declined substantially since 1987, but this overall progress masks persistent and widening geographical and demographical disparities. The burden has increasingly shifted toward rural and western regions, where improvements have lagged. These findings underscore the need for regionally and population-targeted control strategies, particularly for older adults and males in underserved areas.

## CRediT author contribution statement

**Qianru Li:** Conceptualization, Data curation, Methodology, Formal analysis; Writing – original draft. **Siyi He, Mengdi Cao, Yi Teng, Nuopei Tan, Jiachen Wang, Yujie Wu, Tianyi Li, Yuanjie Zheng, Tingting Zuo, Changfa Xia:** Writing – review & editing. **Wanqing Chen:** Conceptualization, Supervision, Funding acquisition, Writing – review & editing.

## Declaration of competing interest

The authors declare that they have no known competing financial interests or personal relationships that could have appeared to influence the work reported in this paper.

## Appendix Supplementary materials

Image, application 1
