# Supplementary material for: Persistent and widening geographic disparities in liver cancer mortality in China, 1987–2021: a 35-year analysis from a national perspective
Source: J Natl Cancer Cent. 2026 Jan 27;6(4):391–9. doi: 10.1016/j.jncc.2025.12.006 (PMC13494644; doi:10.1016/j.jncc.2025.12.006)
Supplement: Supplementary file 1 [file mmc1.pdf]

Supplementary materials

Persistent and widening geographic disparities in liver cancer mortality in China, 1987–2021: a 35-year analysis from a national perspective

Qianru Li, Siyi He, Mengdi Cao, Yi Teng, Nuopei Tan, Jiachen Wang, Yujie Wu, Tianyi Li, Yuanjie Zheng, Tingting Zuo, Changfa Xia, Wanqing Chen

Sensitivity analyses for the 2004 turning point

To confirm that the observed turning point in liver cancer mortality around 2004 was a genuine trend change and not an artifact of the data-source transition, we performed two complementary analyses to assess its robustness.

First, a segmented regression model with automatic breakpoint detection was applied to the 1987–2021 age-standardized mortality rate (ASMR) series. This data-driven analysis consistently identified a significant breakpoint around 2004 (Supplementary Table 4), corresponding to the period of data-source transition.

Second, to further assess robustness, we conducted a series of interrupted time-series (ITS) models using fixed breakpoints for candidate years from 1999 to 2006. This approach tests whether the inflection was attributable to the data-source transition rather than a real epidemiologic change. The ITS model was specified as:

$$\log(ASMR_t) = \beta_0 + \beta_1 Time_1 + \beta_2 D_t + \beta_3 (Times_t - T) D_t + \varepsilon_t$$

where D_t is a post-interruption indicator ($D_t = 1$ if $t \geq T$, 0 otherwise), β_1 represents the pre-interruption trend, β_2 the immediate level change at the breakpoint, and β_3 the change in slope after the breakpoint. This sensitivity analysis yielded consistent results: regardless of the specific breakpoint year tested (1999–2006), the change in slope after the breakpoint (β_3) was always highly significant ($P <$ 0.001; Supplementary Table 5), confirming the robustness of this turning point.

Supplementary Table 1
Annual average percent changes of liver cancer mortality rates in different locations during 1987-2000, overall and by sex.

	Both (95% CI)	*P*-value	Female (95% CI)	*P*-value	Male (95% CI)	*P*-value
**CMR**						
Nationwide	1.69 (1.27, 2.11)	< 0.001	1.78 (1.43, 2.12)	< 0.001	1.68 (1.19, 2.17)	< 0.001
Urban	1.34 (0.81, 1.88)	< 0.001	1.43 (1.01, 1.85)	< 0.001	1.33 (0.70, 1.97)	< 0.001
Rural	2.13 (1.47, 2.79)	< 0.001	2.20 (1.43, 2.98)	< 0.001	2.12 (1.44, 2.80)	< 0.001
**ASMR**						
Nationwide	0.33 (-0.10, 0.77)	0.136	0.18 (-0.14, 0.51)	0.264	0.52 (0.03, 1.02)	0.038
Urban	-0.44 (-0.92, 0.04)	0.075	-0.58 (-0.92, -0.23)	0.001	0.00 (-0.60, 0.61)	0.994
Rural	1.00 (0.32, 1.67)	0.004	1.21 (0.57, 1.85)	< 0.001	1.04 (0.37, 1.71)	0.002

Abbreviations: ASMR, age-standardized mortality rates; CI, confidence interval; CMR, crude mortality rates.

Supplementary Table 2

Summary of interaction term analyses for sex-, urban–rural-, and region-specific trends in liver cancer mortality, China.[*]

Model type	Comparison	Time period	Stratification	*P* value
Segmented regression[a]	Sex × Year	1987-2021	Urban-rural: all	< 0.001
	Sex × Year	1987-2021	Urban-rural: rural	< 0.001
	Sex × Year	1987-2021	Urban-rural: urban	< 0.001
	Urban-rural × Year	1987-2021	Sex: male	< 0.001
	Urban-rural × Year	1987-2021	Sex: female	< 0.001
	Urban-rural × Year	1987-2021	Sex: both	< 0.001
	Sex × Year	2004-2021	Region: central region	< 0.001
	Sex × Year	2004-2021	Region: eastern region	< 0.001
	Sex × Year	2004-2021	Region: western region	< 0.001
	Region × Year	2004-2021	Sex: male	< 0.001
	Region × Year	2004-2021	Sex: female	< 0.001
	Region × Year	2004-2021	Sex: both	< 0.001
Poisson regression[b]	Sex × Year	1987-2021	Urban-rural: all	< 0.001
	Sex × Year	1987-2021	Urban-rural: rural	< 0.001
	Sex × Year	1987-2021	Urban-rural: urban	0.001
	Urban-rural × Year	1987-2021	Sex: male	< 0.001
	Urban-rural × Year	1987-2021	Sex: female	< 0.001
	Urban-rural × Year	1987-2021	Sex: both	< 0.001
APC analysis[c]	Sex × Period	2004-2021	All regions	< 0.001
	Urban-rural × Period	2004-2021	Sex: both	< 0.001
	Region × Period	2004-2021	Sex: both	< 0.001

[*] This table summarizes the results of interaction term tests assessing whether temporal trends in liver cancer mortality significantly differed across sex, urban–rural residence, and geographic regions.

[a] Segmented regression: Models were fitted on age-standardized mortality rates to identify differences in time trends between subgroups over the entire period (1987–2021) and the DSP period (2004–2021).

[b] Poisson regression: Log-linear Poisson models were used to evaluate interactions between calendar year and population subgroups.

[c] APC analysis: APC models were applied to DSP data (2004–2021) to test whether period effects differed by sex, urban–rural residence, or region.

Abbreviations: APC, age–period–cohort; DSP, Disease Surveillance Points.

Supplementary Table 3

Average annual percent changes in liver cancer mortality rates in different regions of China from 1987 to 2021, overall and by sex, excluding data from 2000 and 2004.

	Both (95% CI)	*P*-value	Female (95% CI)	*P*-value	Male (95% CI)	*P*-value
**CMR**						
Nationwide	0.76 (0.60, 0.92)	< 0.001	0.62 (0.43, 0.81)	< 0.001	0.63 (0.45, 0.81)	< 0.001
Urban	0.40 (0.18, 0.62)	< 0.001	0.40 (0.15, 0.65)	0.002	0.44 (0.22, 0.66)	< 0.001
Rural	0.88 (0.66, 1.10)	< 0.001	0.61 (0.36, 0.86)	< 0.001	0.98 (0.75, 1.21)	< 0.001
**ASMR**						
Nationwide	-1.11 (-1.27, -0.94)	< 0.001	-1.36 (-1.55, -1.18)	< 0.001	-1.11 (-1.27, -0.96)	< 0.001
Urban	-1.34 (-1.58, -1.10)	< 0.001	-1.49 (-1.73, -1.25)	< 0.001	-1.28 (-1.52, -1.05)	< 0.001
Rural	-1.22 (-1.43, -1.01)	< 0.001	-1.51 (-1.74, -1.28)	< 0.001	-1.11 (-1.32, -0.89)	< 0.001

Abbreviations: ASMR, age-standardized mortality rates; CI, confidence interval; CMR, crude mortality rates.

Supplementary Table 4

Estimated breakpoints and AAPC before and after each turning point identified by segmented regression from 1987 to 2021. *

Sex	Region	Breakpoint	$AAPC_{Before}$	$AAPC_{After}$
Both	All	2004	0.46	-2.71
Male	All	2005	0.49	-2.57
Female	All	2004	0.37	-3.03
Both	Urban	2004	-0.10	-2.58
Both	Rural	2002	0.88	-2.93

* The breakpoint represents the estimated year of a significant change in mortality trend. $AAPC_{Before}$ and $AAPC_{After}$ denote the average annual percent change before and after the breakpoint, respectively.

Abbreviations: AAPC, average annual percent change; ASMR, age-standardized mortality rate.

Supplementary Table 5

IITS analysis of liver cancer mortality trends in China, 1987–2021, under different breakpoint assumptions.

Breakpoint	β Time	P Time	β Interruption	P Interruption	β Time After	P Time After
1999	0.00	0.86	0.14	0.01	-0.02	< 0.001
2000	0.00	0.54	0.14	0.01	-0.03	< 0.001
2001	0.00	0.38	0.15	0.00	-0.03	< 0.001
2002	0.00	0.38	0.12	0.00	-0.03	< 0.001
2003	0.00	0.38	0.09	0.01	-0.03	< 0.001
2004	0.00	0.38	0.06	0.07	-0.03	< 0.001
2005	0.00	0.08	-0.02	0.71	-0.03	< 0.001
2006	0.01	0.01	-0.08	0.05	-0.03	< 0.001

Abbreviations: ASMR, age-standardized mortality rate; ITS, interrupted time-series.

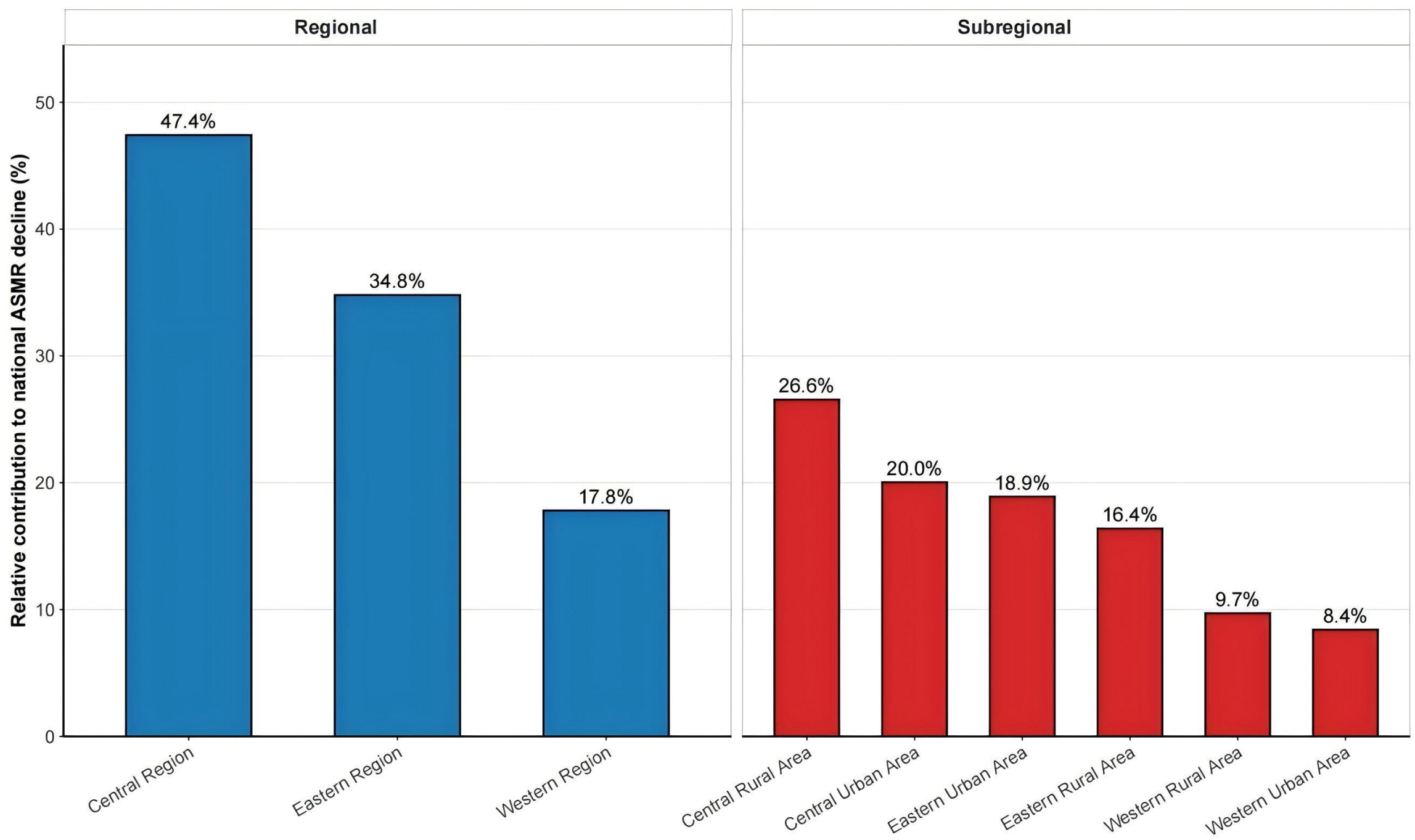

Supplementary Fig.1. Relative contribution of each region to the national decline in ASMR of liver cancer from 2004 to 2021. ASMR, age-standardized mortality rate.

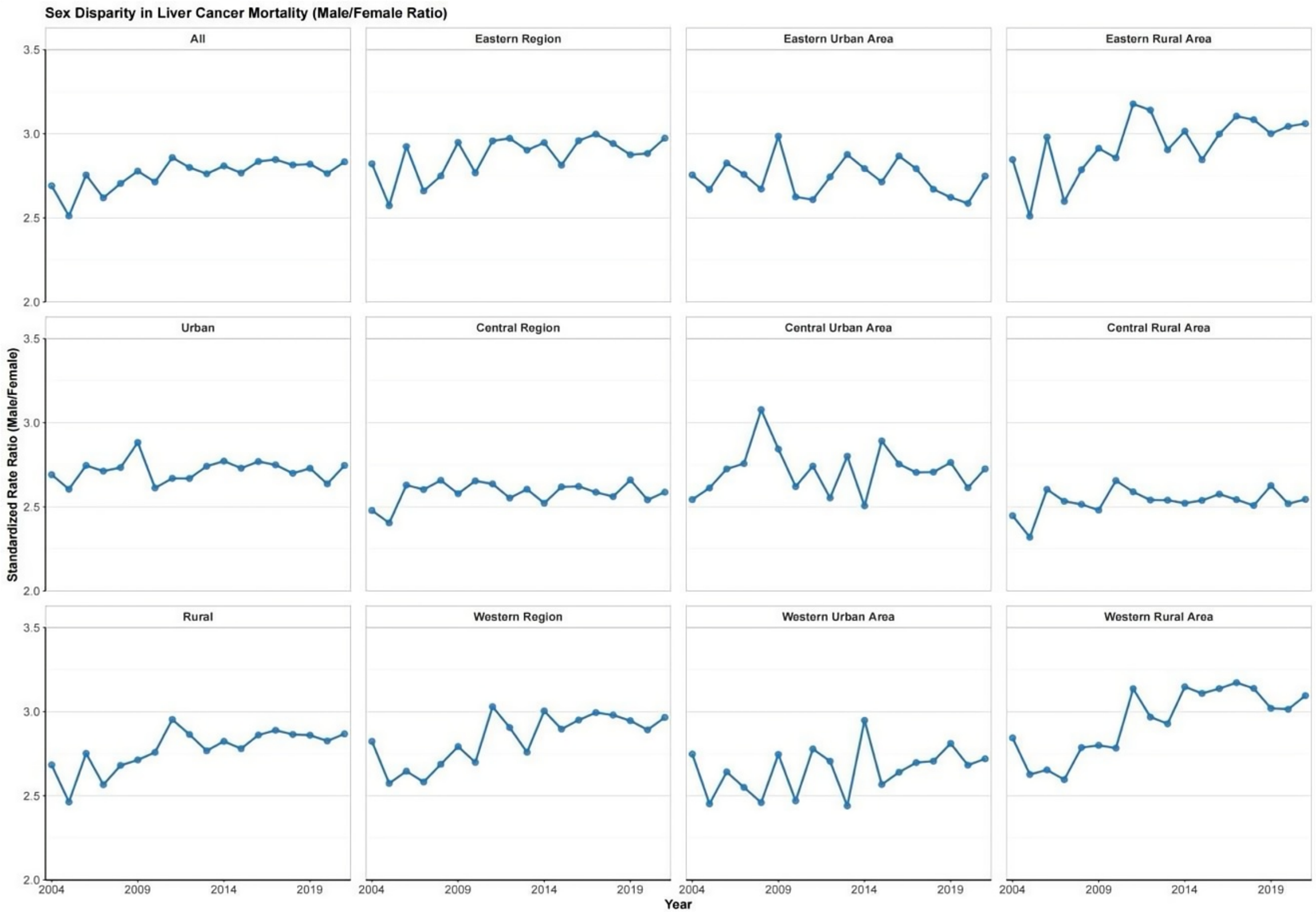

Supplementary Fig.2. Standardized rate ratios of age-standardized liver cancer mortality comparing males vs females, 2004–2021, stratified by region and area.

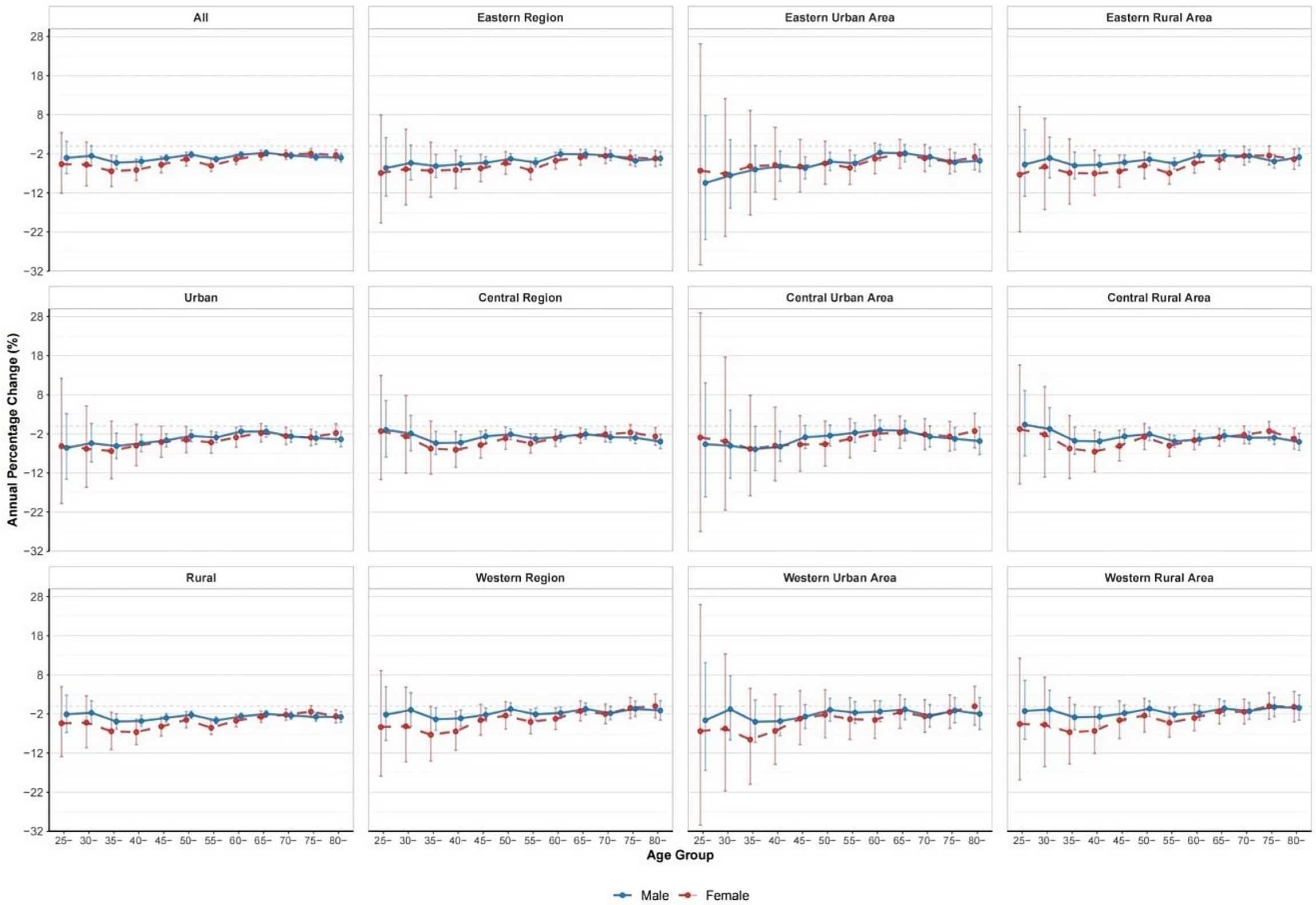

Supplementary Fig.3. Age-specific annual average percent changes of liver cancer mortality rates by sex, regions, and areas during 2004-2021.

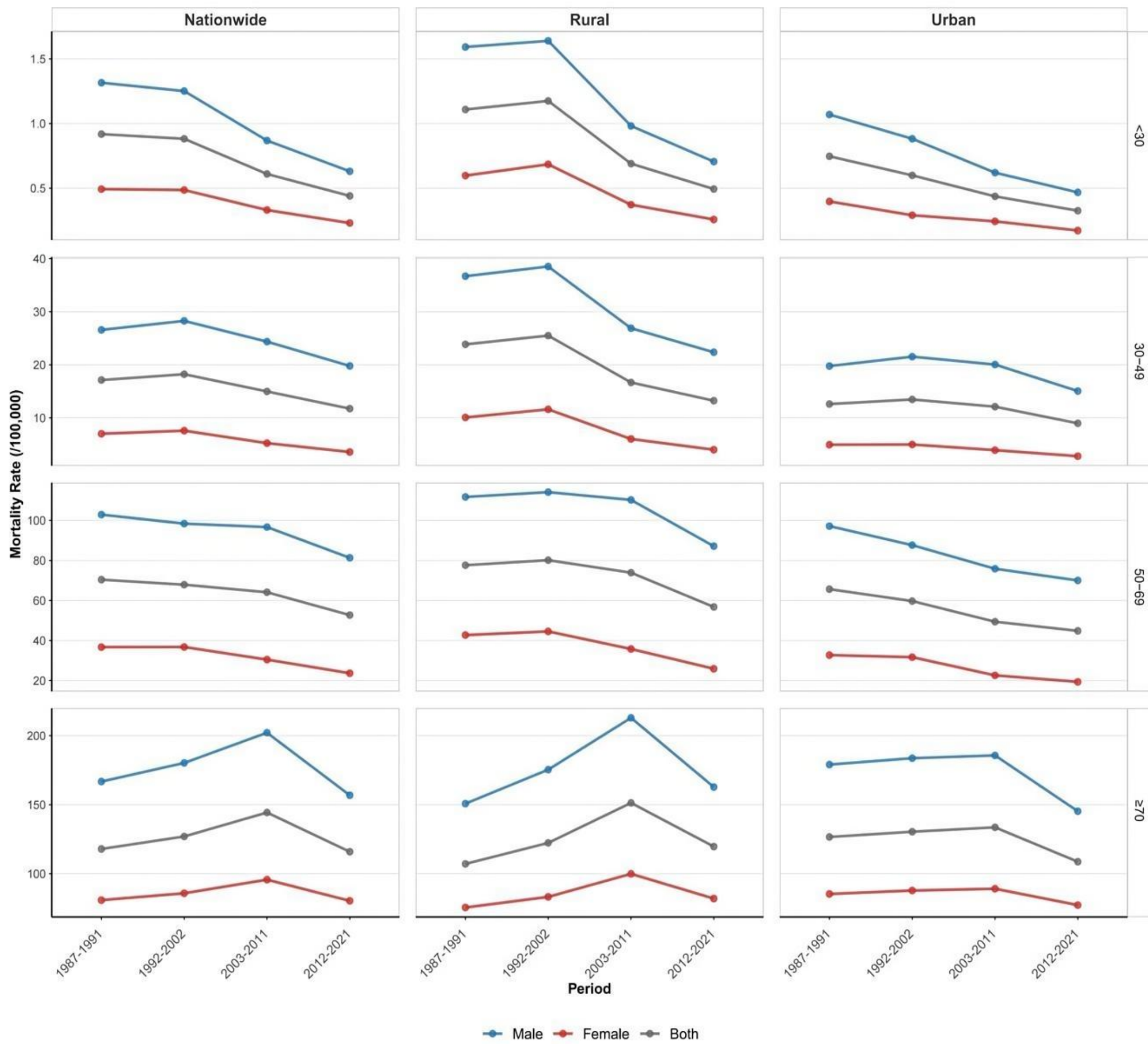

Supplementary Fig.4. Liver cancer mortality rates across different time periods and age groups from 1987 to 2021, stratified by region and sex.

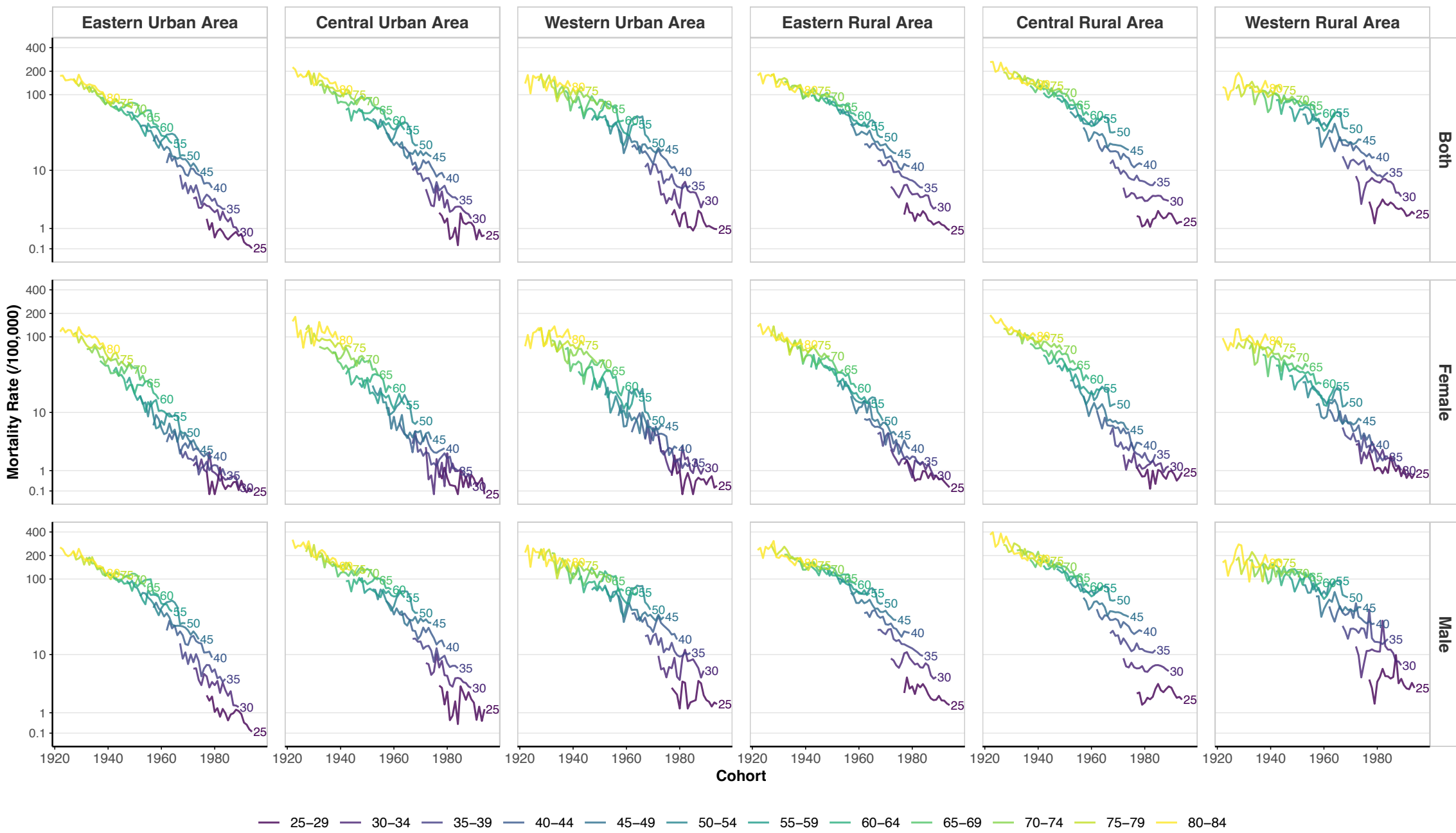

Supplementary Fig.5. Cohort-specific mortality rates of liver cancer on a semilogarithmic scale in different age groups, stratified by sex and areas.
